# Meta-UNet: enhancing skin-lesion segmentation with multimodal feature integration and uncertainty estimation

**DOI:** 10.1007/s11548-025-03490-2

**Published:** 2025-07-30

**Authors:** O. K. Sikha, Alaysia Leilani B. Stone, Miguel A. González Ballester

**Affiliations:** 1 Department of Engineering, Universitat Pompeu Fabra, Barcelona, Spain; 2 MIT Department of Electrical Engineering and Computer Science, Massachusetts Institute of Technology (MIT), Cambridge, MA 02139 USA; 3 ICREA, Barcelona, Spain

**Keywords:** Multimodal U-Net, Uncertainty, Skin cancer, Segmentation

## Abstract

**Purpose:**

Medical image segmentation plays a crucial role in diagnostic pipelines. This study investigates the integration of lesion-specific metadata with image data to enhance segmentation accuracy and reduce predictive uncertainty.

**Methods:**

The standard U-Net architecture was modified to incorporate lesion-specific metadata (Meta-UNet). Various integration strategies, including addition, weighted addition, and embedding layers, were evaluated. Additionally, a Bayesian Meta-UNet with Monte Carlo Dropout (MCD) was developed to assess the impact of metadata integration on model uncertainty. Uncertainty was quantified using measures such as Confidence Maps, Entropy, Mutual Information, and Expected Pairwise Kullback–Leibler divergence (EPKL). An aggregation strategy was also introduced to provide a single comprehensive uncertainty score per image.

**Results:**

Meta-UNet outperformed standard U-Net across PH2, ISIC 2018, and HAM10000 datasets. On PH2, it achieved 84.64% accuracy and 90.62% Intersection over Union (IoU), compared to 83.36% and 89.19%. On ISIC 2018, U-Net scored 71.02 ± 6.69 IoU and 79.89 ± 5.09 Dice. On HAM10000, Meta-UNet achieved 88.66 ± 6.09 IoU and 93.42 ± 5.19 Dice. Meta-UNet reduced uncertainty (e.g., 0.149 vs. 0.1745), highlighting the benefit of metadata integration in improving segmentation accuracy and model confidence.

**Conclusion:**

Integrating lesion-specific metadata into the U-Net architecture significantly improves segmentation accuracy and reduces predictive uncertainty. The inclusion of metadata enhances model confidence and reliability, underscoring its potential to strengthen diagnostic segmentation pipelines.

## Introduction

Accurate segmentation of medical images is essential for diagnosis and treatment planning [1], enabling automated identification of organs, tumors, etc. However, current models face challenges in handling anatomical complexity, ambiguous boundaries, and low-quality images, limiting their clinical reliability [2]. Critically, most models lack uncertainty quantification, making it difficult to assess prediction confidence and determine when human review is needed. Moreover, integrating metadata—such as lesion type or clinical context—can boost accuracy and reduce uncertainty, particularly in complex scenarios. Although studies highlight the potential of metadata-aware segmentation, research in this area remains limited [3]. The metadata used in this study includes various histological and morphological features of the lesion. An uncertainty analysis technique, Monte Carlo Dropout (MCD), is employed to quantify uncertainty and assess the impact of metadata on reducing uncertainty in skin cancer segmentation. The major contributions of the proposed work are as follows:

- Proposed **Meta-UNet**, an enhanced segmentation model that integrates medical metadata with traditional U-Net architecture to improve the accuracy and reliability of skin cancer segmentation. By incorporating lesion-specific features, Meta-UNet effectively reduces uncertainty in segmentation, particularly for complex cases.
- A comprehensive quantitative and qualitative evaluation of the proposed Meta-UNet segmentation model on three publicly available datasets: PH2, ISIC 2018 and HAM10000. This evaluation includes a comparison with the standard U-Net model and other state-of-the-art (SoTA) models, focusing on key metrics such as Intersection over Union (IoU) and Dice score to assess segmentation accuracy. Various metadata integration strategies were also examined to assess their effect on segmentation performance.
- Introduced a Bayesian adaptation of the standard U-Net, as well as the proposed Meta-UNet using MCD combined with various uncertainty estimates. An aggregation strategy was also proposed to consolidate these measures into a single uncertainty score per image. The results show that integrating metadata into Meta-UNet improves model confidence and yields more reliable, interpretable predictions compared to standard U-Net.

The paper is structured as follows: section “Related works” reviews related work; section “Meta-UNet: multimodal U-Net for segmentation” details the proposed multimodal U-Net; section “Dataset” Sect describes the datasets; section “Implementation details” covers implementation; section “Experiments and results” presents results; and section “Conclusion and future work” concludes with future directions.

## Related works

**CNN-Based Models:** Deep Neural Networks (DNNs) and Convolutional Neural Networks (CNNs) have become standard for skin lesion segmentation [4, 5]. U-Net is widely used for medical image segmentation [6], with many variants [7–9] including deeper layers and contextual information. Attention mechanisms have been introduced, such as attention gates [10], multi-resolution attention [11], and other feature-based methods [12–14]. To address segmentation ambiguity, uncertainty-aware models like Bayesian U-Net [15] generate uncertainty maps alongside predictions. Additionally, metadata integration has shown potential to improve segmentation reliability. For instance, Dong et al. [16] introduced the Cross-Modality Collaborative Feature Exploration (CMC) module to exploit relationships between dermoscopic images and clinical metadata. Kendall and Gal [17] demonstrated that metadata can improve performance in uncertain cases. However, this field is less explored than U-Net innovations.

*Transformer-Based Models:* Transformers, initially introduced for machine translation [18], have achieved SoTA performance in various NLP tasks. Many computer vision-focused modifications have been proposed. Chen et al. [19] introduced TransUNet, combining the strengths of Transformers and U-Net for medical image segmentation. It uses a CNN backbone to extract feature maps, which are tokenized and processed by a Transformer encoder for capturing global context, while the decoder upsamples and integrates high-resolution CNN features for precise localization. Similarly, Cao et al. [20] proposed Swin-Unet, a pure Transformer-based U-shaped architecture with skip connections for effective local–global semantic learning. In the domain of skin lesion segmentation, [21] showed that TransUNet outperforms traditional models in terms of accuracy and Dice score, highlighting the synergy between CNNs and Transformers. Additionally, [22] developed the Boundary-Aware Transformer (BAT), designed to enhance segmentation by emphasizing lesion boundaries. Table 1 summarizes the relevant literature.

**Table 1:** Literature survey on segmentation models for medical imaging

Author/Year	Data type	Model architecture	Working principle
Ronneberger et al. [6]	Image	U-Net	Encoder-decoder with skip connections
Gal and Ghahramani [15]	Image	Bayesian U-Net	Dropout for uncertainty estimation
Milletari et al. [9]	Image	V-Net	3D U-Net with residual connections
Kendall and Gal [17]	Multimodal	Bayesian CNN	Uses metadata for uncertainty handling
Kakeya et al. [8]	Image	U-JapaNet	U-Net variant for Japanese datasets
Oktay et al. [10]	Image	Attention U-Net	Attention gates focus on relevant features
Yagi et al. [7]	Image	U-Net variant	Context-aware layers for abdominal segmentation
Sinha and Dolz [11]	Image	Multi-resolution Attention U-Net	Aggregates attention maps from multiple resolutions
Zhang et al. [14]	Image	Context-aware CNN	Merges spatial and contextual attention
Zhou et al. [23]	Image	U-Net++	Improved skip connections for multiscale features
Jha et al. [24]	Image	ResUNet++	Dense skip connections for accurate segmentation
Gu et al. [12]	Image	Attention CNN	Combines channel and spatial attention
Jha et al. [4]	Image	DoubleU-Net	Stacked U-Nets for coarse-to-fine segmentation
Isensee et al. [25]	Image	nnU-Net	Self-configuring U-Net
Li et al. [13]	Image	Transformer-inspired U-Net	Combines CNN and transformer-based attention
Chen et al. [19]	Image	TransUNet	Combines CNN encoder with Transformer decoder
Wang et al. [22]	Image	BAT (Boundary-Aware Transformer)	Focuses on boundary precision with edge attention
Basak et al. [5]	Image	MFSNet	Multi-scale fusion with squeeze-and-excitation
Cao et al. [20]	Image	Swin-Unet	Transformer U-shape with hierarchical attention
Dong et al. [16]	Multimodal	CMC Module + CNN	Fuses clinical and dermoscopic images
Plutenko et al. [26]	Multimodal	Metadata-guided CNN	Uses metadata via multitasking
Perera et al. [21]	Image	Mobile-UNeTr	Hybrid CNN-Transformer for skin lesion segmentation
Gu et al. [27]	Multimodal	MMY-Net	U-Net3+ with text encoder and self-attention
Khan et al. [28]	Multimodal	MLP + CNN	Uses cardiac metadata to condition segmentation network

## Meta-UNet: multimodal U-Net for segmentation

**Fig. 1:**
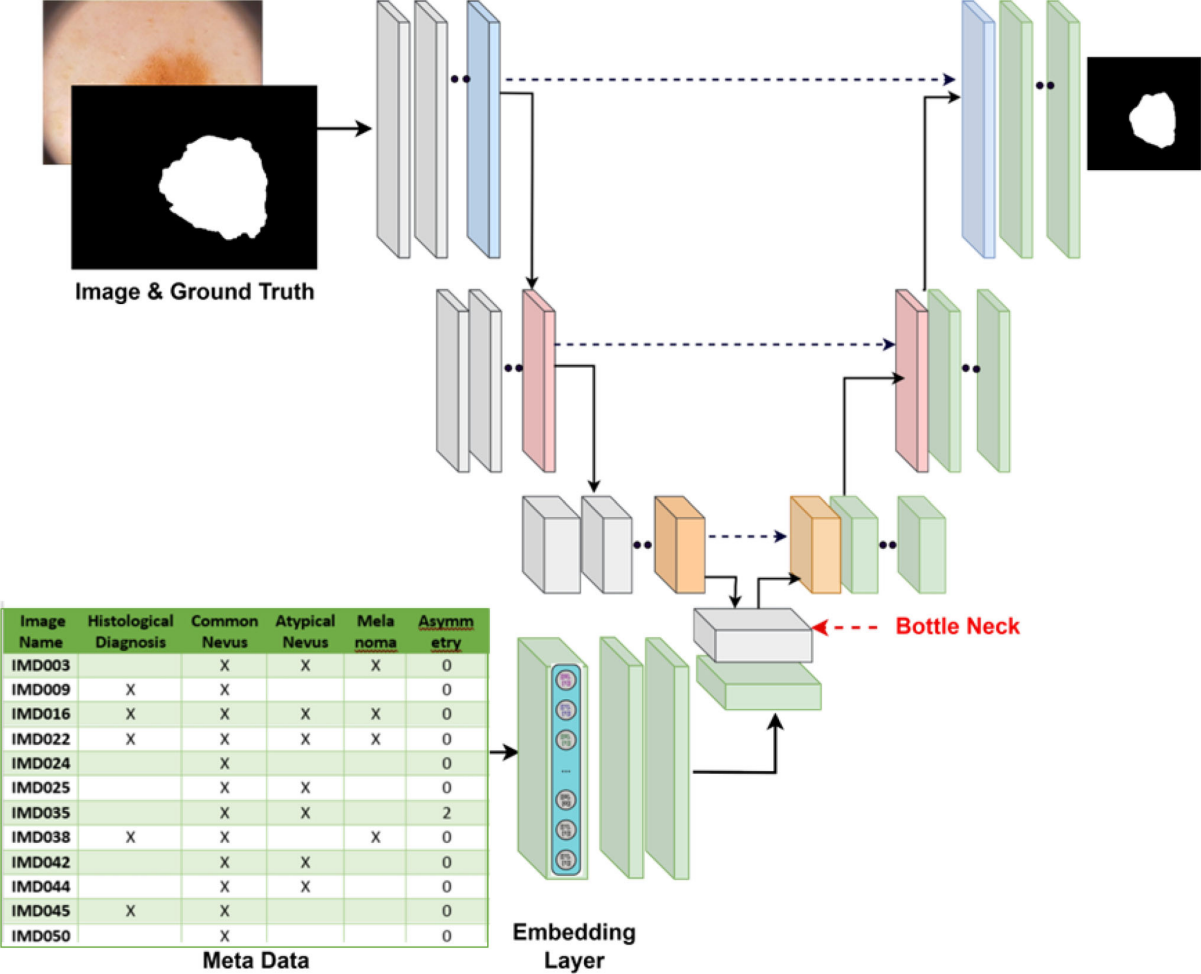
Proposed Meta-UNet architecture augments a standard U-Net backbone with clinical metadata integration for skin lesion segmentation

This section details the proposed Meta-UNet architecture in detail. As illustrated in Fig. 1, Meta-UNet architecture the standard U-Net [6] by incorporating metadata into the image segmentation process. The U-Net backbone processes the input image through an encoding path, consisting of convolutional and max-pooling layers, progressively extracting features and compressing the spatial dimensions. Let $\textbf{I} \in \mathbb {R}^{H \times W \times C}$ be the input image to Meta-UNet. Each encoding block consists of two convolutional layers, denoted as:

1 $$\begin{aligned} \textbf{F}_l=\operatorname {ReLU}\left( \textbf{W}_l * \textbf{F}_{l-1}+\textbf{b}_l\right) \end{aligned}$$

where $\textbf{W}_l \text{ and } \textbf{b}_l$ are the weights and biases of the *l*-th layer, * is the convolution operator, ReLU is the activation function, and $\textbf{F}_{l-1}$ is the feature map from the previous layer. Max pooling downsamples, reducing the spatial dimensions, by selecting the maximum value from each patch of the feature map: $\textbf{F}_l=\operatorname {MaxPool}\left( \textbf{F}_{l-1}, k\right)$, where *k* is the pooling window size (e.g., 2$\times$2). The number of filters doubles with each downsampling step (e.g., 64$\rightarrow$128$\rightarrow$256$\rightarrow$512$\rightarrow$1024) until the latent feature representation $\textbf{F}_{\text{ latent }} \in \mathbb {R}^{7 \times 7 \times 1024}$ is reached, which encodes the global information. Simultaneously, the metadata vector $\textbf{M}$ of size 29 (as shown in the metadata table in Fig. 1) is processed using the embedding layer to map each metadata feature to a dense vector representation. The embeddings are concatenated and further processed through two dense layers with 36 and 49 neurons, respectively, using ReLU activations. The final output is reshaped into a spatial tensor $\textbf{M}_{\text{ embed }} \in \mathbb {R}^{7 \times 7 \times 1}$ to match $\textbf{F}_{\text{ latent }}$.

2 $$\begin{aligned} \textbf{F}_{\text{ combined }}=\text {Concat }\left( \textbf{F}_{\text {latent}}, \textbf{M}_{\text{ embed }}\right) \end{aligned}$$

This fusion integrates global image features with the metadata, allowing context-aware predictions. ${\textbf {F}}_{combined}$ is passed through the decoding path of Meta-UNet, which consists of up-convolutions (UpConv) and skip connections, refining spatial details: $\textbf{F}_l=\operatorname {UpConv}\left( \textbf{F}_{l+1}\right) +$ CropAndCopy $\left( \textbf{F}_{l_{\text{ skip }}}\right)$. The output segmentation map $\textbf{O} \in \mathbb {R}^{H_{\text{ out }} \times W_{\text{ out }} \times C_{\text{ out }}}\left( \right.$e.g., $\left. \textbf{O} \in \mathbb {R}^{224 \times 224 \times 1}\right)$ provides pixel-wise class probabilities. We experimented with different feature fusion techniques to combine the metadata’s transformed features with the latent vector of U-Net. The findings indicate that integrating disease-specific metadata through embedding layers significantly enhances segmentation accuracy, as evidenced by improved IoU and Dice score.

### Bayesian Meta-UNet for uncertainty quantification

To assess how integrating metadata influences the model’s uncertainty and confidence, a Bayesian version of Meta-UNet was developed. In Bayesian learning, the posterior predictive distribution is expressed as:

3 $$\begin{aligned} p(y \mid x, \mathcal {D})=\int p(y \mid x, w) p(w \mid \mathcal {D}) d w \end{aligned}$$

where *D* is the training set, *x* is the input image, *y* is the output label, *w* is the neural network weights, $p(y \mid x, w)$ is the likelihood, and $p(w \mid D, w)$ is the posterior distribution. Full Bayesian inference in deep learning is computationally complex, but it can be approximated using techniques such as MCD. MCD applies a Bernoulli distribution to the weights and minimizes EPKL with a variational distribution. During prediction, *T* forward passes are performed with active dropout layers after each convolutional block, enabling the computation of predictions and uncertainty metrics.

**Table 2:** Summary of skin lesion datasets used for segmentation

Dataset	No. of images	Diagnosis	Visual features captured
ISIC 2018 [32]	$\sim$2600	Melanoma, Nevus, Seborrheic Keratosis	Pigment network, dots/globules, streaks, regression, blue-white veil
PH2 [31]	200	Melanoma, Atypical Nevus, Common Nevus	Pigment network, dots/globules, streaks, regression, blue-white veil
HAM10000 [33]	10,015	7 classes including melanoma, nevus, and others	Pigment network, dots/globules, streaks, regression, blue-white veil

**Table 3:** Segmentation performance comparison of standard U-Net and Meta-UNet using different loss functions

Model	Loss function	Mean IoU (%)	Mean dice (%)
Standard U-Net	BCE	80.59 ± 7.95	88.19 ± 8.50
Standard U-Net	IoU	83.36 ± 7.95	89.19 ± 8.50
Meta-UNet	BCE	81.64 ± 8.40	88.68 ± 8.50
**Meta-Unet**	**IoU**	**84.64 ± 8.40**	**90.62 ± 8.50**

The best model is bolded

**Fig. 2:**
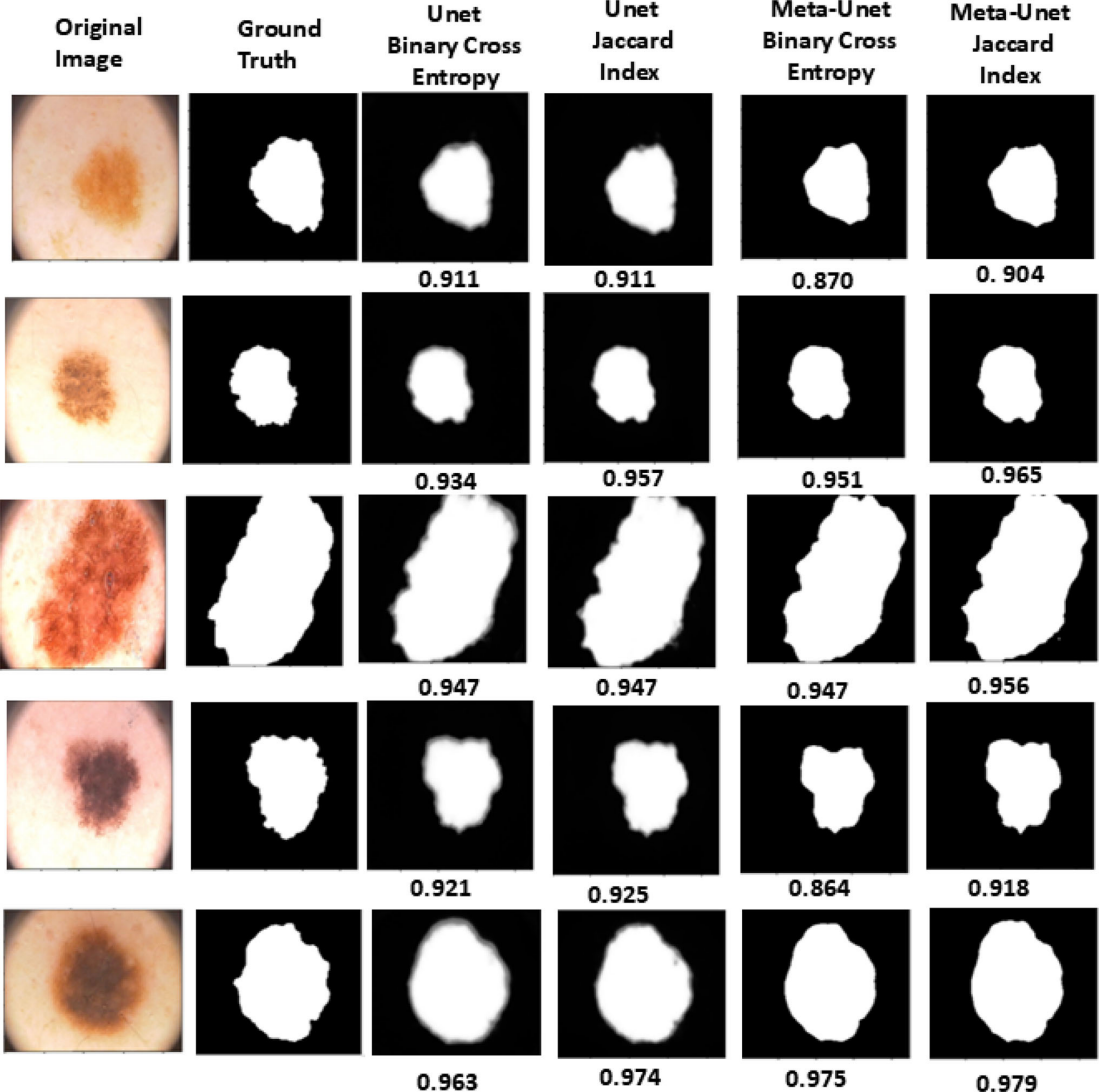
Visual comparison of segmentation outputs from standard U-Net and Meta-UNet trained with different loss functions

**Table 4:** Performance comparison of different multimodal feature integration strategies

Model	Multimodal feature integration strategy	Mean IoU (%)	Mean Dice (%)
Standard U-Net	Baseline model with image features only	80.59	88.19
Meta-UNet #1	One-hot encoding followed by dense layers for metadata integration	81.64	84.10
Meta-UNet #2 (60-40)	Weighted integration (0.6 * Image features + 0.4 * Metadata features)	82.36	86.68
Meta-UNet #2 (70-30)	Weighted integration (0.7 * Image features + 0.3 * Metadata features)	83.16	89.71
Meta-UNet #3 (80-20)	Weighted integration (0.8 * Image features + 0.2 * Metadata features)	82.9	88.8
Meta-UNet #4 (90-10)	Weighted integration (0.9 * Image features + 0.1 * Metadata features)	69.8	75.0
**Meta-UNet #5**	**Embedding layer for metadata integration**	**84**.**64**	**90**.**62**

Best results are bolded

**Table 5:** Comparison of skin segmentation for the PH2 dataset [31] with SoTA segmentation models

Ph2-Dataset [31]		
	Mean IoU	Mean Dice
Standard U-Net [6]	80.59 ±7.95	88.19 ± 8.5
UNet++ [23]*	84.35 ± 8.96	90.25 ± 9.23
AttU-Net [10]*	82.21 ± 9.23	90.03 ± 8.96
SwinUnet [20]*	87.16 ± 8.10	92.88 ± 8.40
TransUNet(ViT) [19]*	90.85	83.97
TransUNet(R50) [19]*	86.76	92.59
Bayesian Unet-MCD	83.16± 9.41	89.1 ± 8.25
**Meta-UNet (Ours)**	**84.64 ± 8.40**	**90.62 ± 8.50**

Model results with “*” are reproduced from the published source code

The bold values represent result obtained from our model. Results of Meta-UNet (Ours) are Highlighted in Bold

**Table 6:** A comparison of skin segmentation for the ISIC-2018 [32] dataset with SoTA segmentation models

ISIC 2018-Dataset [32]		
	Mean IoU	Mean Dice
Standard U-Net[6]	71.02 ± 6.69	79.89 ± 5.09
UNet++ [23]*	85.36 ± 8.10	77.21 ± 8.40
AttU-Net [10]*	85.25 ± 5.85	81.21 ± 7.23
ResUNet [24]*	70.15 ± 9.74	79.15 ± 7.42
SwinUnet [20]*	81.67	88.87
TransUNet (ViT) [19]*	81.67	88.91
TransUNet (R50) [19]*	82.79	89.71
Bayesian UNet-MCD	86.16± 7.07	91.05± 6.17
**Meta-UNet (Ours)**	**88.21± 6.09**	**93.14± 5.19**

Model results with “*” are reproduced from the published source code

The bold values represent result obtained from our model. Results of Meta-UNet (Ours) are Highlighted in Bold

**Table 7:** A comparison of skin segmentation for the HAM10000 Dataset [33] with SoTA segmentation models

HAM10000 Dataset [33]		
	Mean IoU	Mean Dice
Standard U-Net [6]	71.02 ± 6.69	79.89 ± 5.09
DoubleU-Net [4]*	81.2	84.3
SegNet [4]*	82.1	81.6
MFSNet [5]*	90.2	90.6
Bayesian Unet-MCD	88.52± 7.09	93.32± 7.19
Meta-UNet (Ours)	88.66± 6.09	93.42± 5.19

Model results with “*” are reproduced from the published source code

This approach facilitates the simultaneous estimation of predictions and uncertainties, thereby improving the model’s reliability and robustness. To quantify pixel-wise uncertainty we used four uncertainty estimates (with italicized variable names): (1) predictive entropy *H* captures both aleotoric and epistemic uncertainty; (2) confidence map *C* is calculated as the maximum value across all classes *c* over averaged candidate segmentations; (3) Mutual Information *MI* captures the disagreement between predictions, thus capturing the epistemic uncertainty; and (4) EPKL divergence *EPKL* quantifies the average difference between the predicted class probabilities across models and the expected class probabilities. In our experiment, MCD was used with $T = 10$ during prediction to compute uncertainty. To further quantify the impact of metadata integration on uncertainty estimation, we adopt an aggregation strategy proposed in our previous work [29]. This method combines the aforementioned uncertainty metrics into a single aggregate uncertainty score per image, formulated as:

4 $$\begin{aligned} U_{\text{ tot } }=\alpha * C-\beta * H+\gamma * M I-\delta * E P K L \end{aligned}$$

Prior to aggregate score calculation, each uncertainty measure is normalized to ensure comparability across scales. The weight parameters $\alpha$, $\beta$, $\gamma$, and $\delta$ are set to 0.4, 0.2, 0.2, and 0.2, respectively, based on experimental insights from our previous study [29] and supporting literature [30], which underscore the strong relevance of confidence maps in uncertainty quantification. This composite metric provides a more robust evaluation of model performance than any measure in isolation.

**Fig. 3:**
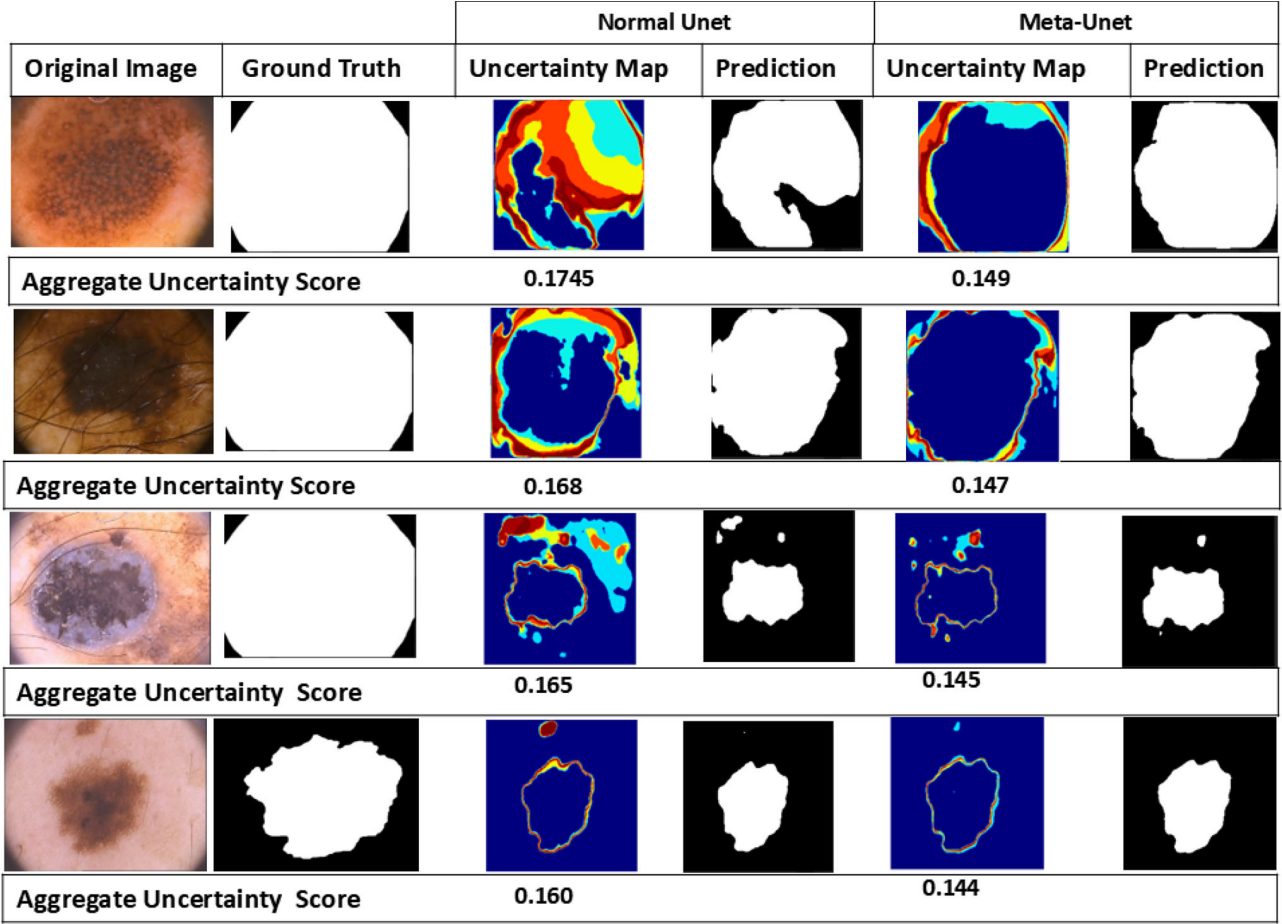
Comparison of uncertainty maps and predictions: standard U-Net versus Meta-UNet

**Fig. 4:**
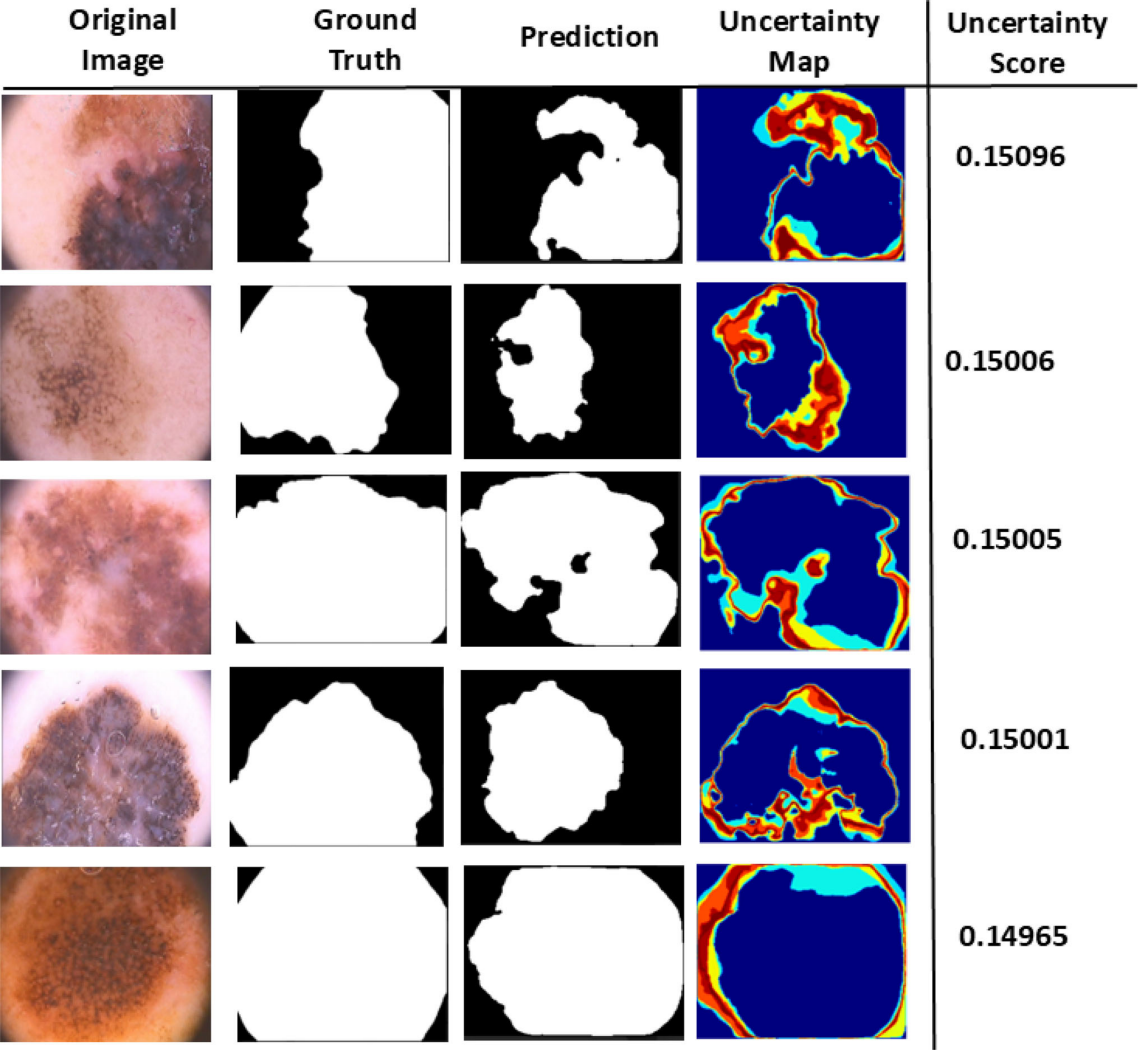
Top five images from the test set with highest aggregated uncertainty scores and corresponding model predictions)

## Dataset

To evaluate the proposed Meta-UNet, we used three publicly available skin lesion segmentation datasets: PH2 [31], ISIC 2018 [32], and HAM10000 [33]. Each dataset includes dermoscopic images, ground truth masks, and accompanying metadata. All images were resized to 224$\times$224 pixels and split into training, validation, and testing sets in a 70:10:20 ratio. The metadata includes histopathological labels (e.g., melanoma, blue nevus) and dermoscopic features (e.g., pigment network, streaks, blue-whitish veil). This combination supports both segmentation and classification tasks. Table 2 provides a summary of the datasets.

## Implementation details

The proposed Meta-UNet was implemented in TensorFlow using the Keras API and trained with an Intel CPU and NVIDIA RTX 3090 GPU. All models used the Adam optimizer with a learning rate of 1e-4, batch size of 16, and were trained for 200 epochs. A ModelCheckpoint callback was employed to save the best model based on validation loss. Early stopping with a patience of 20 epochs was applied to avoid overfitting. Hyperparameter tuning was performed through grid search on the validation set. Image inputs (224$\times$224$\times$3) were preprocessed and augmented using TensorFlow’s standard augmentation layers (e.g., flips, rotations, scaling). The associated metadata was simultaneously augmented to match the transformed image, ensuring consistent input across modalities. For Bayesian inference, we used 10 stochastic forward passes with dropout active during inference (MCD), enabling uncertainty estimation.

**Fig. 5:**
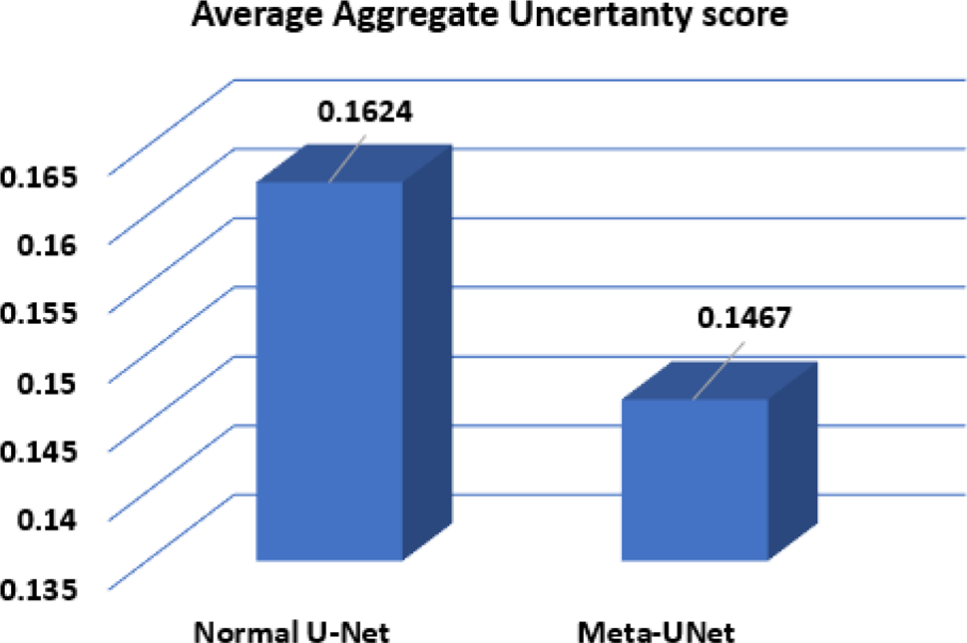
Comparative analysis of average aggregate uncertainty scores for standard U-Net and Meta-UNet models

## Experiments and results

We evaluate the performance of the proposed Meta-UNet compared to standard U-Net and other SoTA segmentation models in terms of IoU and Dice score. Additionally, we assess how metadata integration at the U-Net bottleneck affects the model’s pixel-wise uncertainty.

### Loss function comparison in lesion segmentation

This section compares the segmentation performance of the proposed Meta-UNet and standard U-Net using two loss functions: Binary Cross Entropy (BCE) and IoU. The performance metrics include Mean IoU and Mean Dice score, which provide a comprehensive assessment of the models’ segmentation accuracy and overlap quality. The results of the comparison are summarized in Table 3, which highlights the superior performance of Meta-UNet in all evaluated metrics. Using BCE as the loss function, Meta-UNet achieves slightly higher Mean IoU and Mean Dice, demonstrating the benefit of metadata integration. The performance gap widens with IoU as the loss function, where Meta-UNet achieves a significantly higher Mean IoU (84.64%) and Mean Dice coefficient (90.62%) compared to standard U-Net (80.59% and 88.19%, respectively). These findings confirm that integrating metadata into the U-Net bottleneck improves contextual understanding and segmentation accuracy.

Figure 2 shows the predicted segmentation maps from the standard U-Net and proposed Meta-UNet, trained with different loss functions as detailed in Table 3. The Dice scores below each predicted mask provide a quantitative assessment of segmentation performance. The results demonstrate that Meta-UNet consistently produces segmentation maps more aligned with the ground truth than U-Net, especially when trained with the IoU loss function.

### Comparison of different multimodal feature integration strategies

To evaluate multimodal feature integration strategies, we tested several models that combine image features at the U-Net bottleneck with metadata. These strategies include: (1) appending metadata processed through dense layers, (2) weighted addition of processed metadata at different ratios, and (3) using embedding layers for concatenation. Table 4 summarizes the results. Meta-UNet #5, which uses embedding layers for metadata, achieved the best results (Mean IoU 84.64%, Dice 90.62%), highlighting the advantage of embedding-based fusion. Overall, balanced weighted integration (e.g., 70-30) and embeddings proved most effective in enhancing segmentation via metadata.

### Comparison with state-of-the-art segmentation models

This section compares the proposed Meta-UNet with other SoTA segmentation models, including both traditional and Transformer-based models, on three public datasets.

#### Results on PH2 dataset

Table 5 compares Meta-UNet with several SoTA models on the PH2 dataset. Meta-UNet achieves a Mean IoU of 84.64 ± 8.40 and Dice score of 90.62 ± 8.50, outperforming standard U-Net, AttU-Net, and UNet++. While transformer-based models like TransUNet (e.g., ViT variants often exceed 100 M parameters) offer strong performance, they come with high computational costs. In contrast, Meta-UNet achieves competitive or better results with just 31 M parameters, highlighting its efficiency and accuracy.

**Fig. 6:**
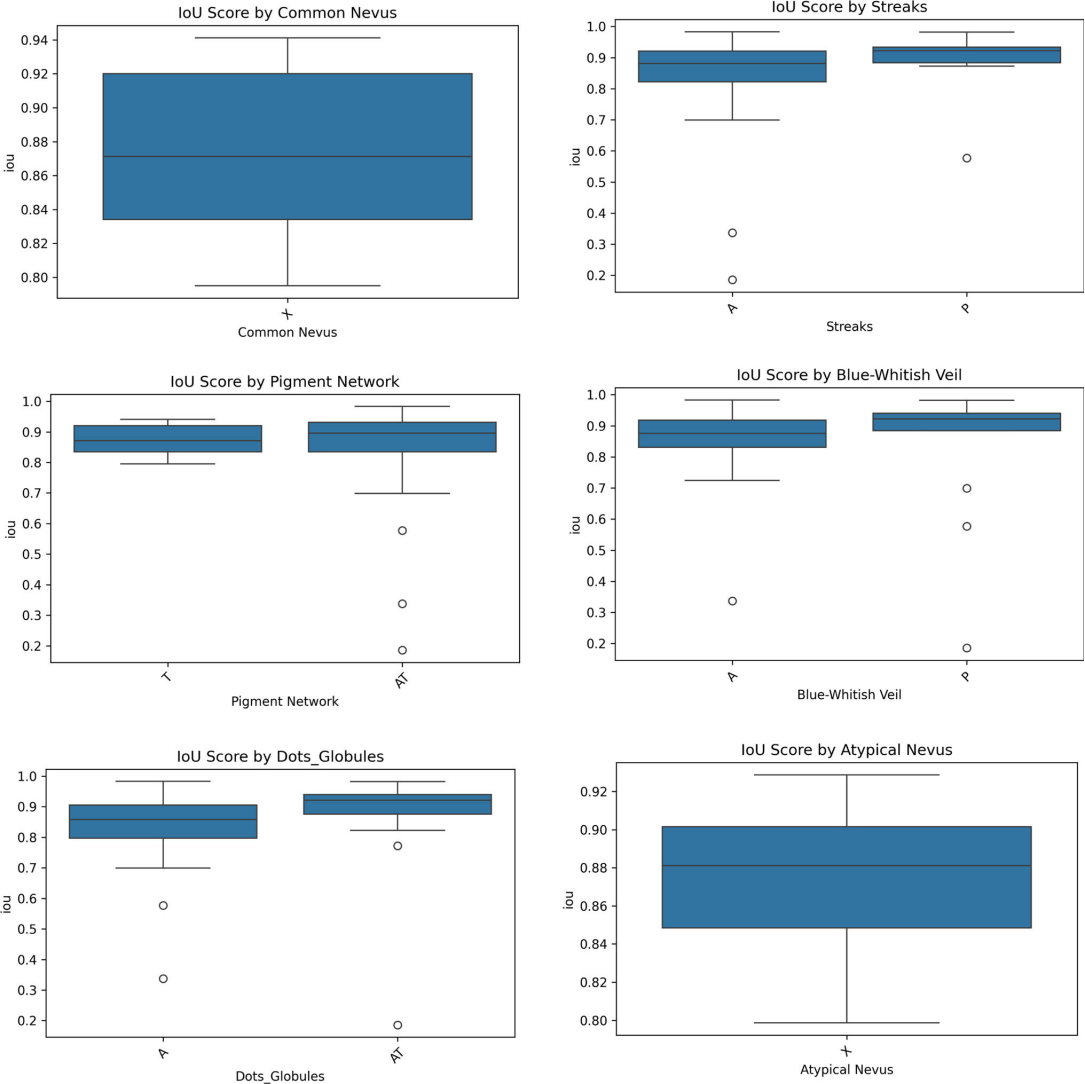
Variation in segmentation performance (IoU) across lesion metadata attributes on PH2 dataset)

#### Results on ISIC dataset

On the ISIC-2018 [32] dataset, the proposed Meta-UNet achieves SoTA segmentation performance, significantly outperforming both traditional and advanced deep learning models. As shown in Table 6, Meta-UNet achieves the highest performance among all evaluated models, with a Mean IoU of 88.21 ± 6.09 and a mean Dice score of 93.14 ± 5.19. Bayesian UNet-MCD and the TransUNet models also perform quite well. These results show that adding metadata helps Meta-UNet understand the image better, leading to more accurate segmentation.

#### Results on HAM10000 dataset

On the HAM10000 dataset [33], Meta-UNet shows strong and consistent performance, achieving a Mean IoU of 88.66 ± 6.09 and a mean Dice score of 93.42 ± 5.19, as shown in Table 7. Bayesian UNet-MCD and MFSNet have competitive, but lower IoU and Dice scores. The relatively low variance in Meta-UNet’s results highlights its robustness and reliability when applied to diverse dermoscopic images, confirming its ability to generalize well across datasets.

### Metadata-driven uncertainty reduction and quantitative confidence analysis

This section highlights how integrating lesion-specific metadata into Meta-UNet enhances prediction confidence and reduces uncertainty. As illustrated in Fig. 3, uncertainty maps reveal pixel-level prediction confidence, where warmer colors denote higher uncertainty. Compared to the standard U-Net, Meta-UNet exhibits significantly fewer high-uncertainty regions—especially around challenging boundaries—resulting in smoother, more accurate segmentations. For instance, in the third row of Fig. 3, Meta-UNet’s uncertainty map is predominantly blue, indicating greater model confidence.

To quantitatively evaluate this improvement, we use an aggregated uncertainty score (defined in Eq. 4) that summarizes multiple uncertainty measures into a single value per image. Meta-UNet consistently achieves lower scores (e.g., 0.149 vs. 0.1745 in the first row of Fig. 3), reflecting reduced ambiguity and improved alignment with ground truth. Furthermore, ranking images by this score—as shown in Fig. 4—helps identify those with noisy or ambiguous labels. High-scoring images often exhibit substantial disagreement between predictions and ground truth, commonly due to annotation errors or lesion boundary ambiguity. Figure 5 presents the average aggregate uncertainty scores across the test set. Meta-UNet achieves a lower mean score ($\sim 0.145$) compared to standard U-Net ($\sim 0.165$), reinforcing its effectiveness in producing more reliable and confident predictions. This demonstrates the value of metadata-aware uncertainty modeling not only for improving segmentation accuracy but also for identifying problematic cases that may benefit from re-annotation.

### Impact of lesion metadata on segmentation performance

Figure 6 illustrates the variation in segmentation performance (IoU scores) across different lesion characteristics on PH2 dataset, highlighting the influence of metadata on model accuracy. Lesions exhibiting features such as pigment networks, dots/globules, or classified as common nevi tend to achieve higher and more consistent IoU scores, suggesting that these structural patterns provide reliable and interpretable visual cues that facilitate precise boundary detection. In contrast, the presence of streaks and blue-whitish veils introduces greater variability in IoU, with several low-performing outliers, indicating that these features may obscure lesion boundaries or exhibit inconsistent visual representations. Atypical nevi also display a wider spread in performance compared to common nevi, likely due to their heterogeneous morphology. These findings underscore that certain dermatological patterns contribute to more confident model predictions, while others pose segmentation challenges due to visual complexity or ambiguity.

## Conclusion and future work

This work introduced Meta-UNet, an enhanced version of U-Net that integrates lesion-specific metadata to improve medical image segmentation. By combining image data with contextual metadata, Meta-UNet outperforms the standard U-Net in both segmentation accuracy and IoU across three public datasets, while also reducing predictive uncertainty. Uncertainty quantification using methods like MCD revealed consistently lower uncertainty scores for Meta-UNet, indicating higher confidence and reliability. An aggregation strategy further highlighted Meta-UNet’s advantage, with a reduced aggregate uncertainty score (0.149 vs. 0.1745). These findings underscore the value of metadata in improving segmentation dependability in clinical settings. Future work will explore advanced fusion techniques, diverse metadata types, and broader validation across imaging modalities to strengthen generalizability.
